# Democratic Systems Increase Outgroup Tolerance Through Opinion Sharing and Voting: An International Perspective

**DOI:** 10.3389/fpsyg.2018.02151

**Published:** 2018-11-13

**Authors:** Fei Hu, I-Ching Lee

**Affiliations:** ^1^Department of Psychology, National Chengchi University, Taipei, Taiwan; ^2^Department of Psychology, National Taiwan University, Taipei, Taiwan

**Keywords:** democratic systems, opinion sharing, voting, outgroup tolerance, intergroup relationship

## Abstract

Democracy may contribute to friendly attitudes and positive attitudes toward outgroups (i.e., outgroup tolerance) because members of democratic societies learn to exercise their rights (i.e., cast a vote) and, in the process, listen to different opinions. Study 1 was a survey study with representative samples from 33 countries (*N* = 45,070, 53.6% female) and it showed a positive association between the levels of democracy and outgroup tolerance after controlling for gender, age and the rate of immigrants influx from 2010 to 2013. Study 1 demonstrated that members in countries with higher political participation and civil liberty showed greater tolerance toward immigrants. In Study 2, we conducted an experimental study in Taiwan (*N* = 93, 67.7% female) to further examine two potential mediators (opinion sharing and voting) of the effect of democratic system on tolerance toward outgroups (i.e., attitudes toward mental patients) after controlling for gender and age. We found that when individuals were allowed to share opinions *and* vote, they had the highest positive other-oriented emotions toward mental patients, which in turn led to greater tolerance toward outgroups compared to those who were not allowed to share opinions or vote. In general, these results demonstrated that the democratic system plays a critical role in increasing outgroup tolerance. Limitations of the two studies and implications regarding opinion sharing, voting, democratic systems, and effects on outgroup tolerance are discussed.

## Introduction

Promoting social tolerance and fostering acceptance of diverse outgroups has been a challenging task in modern societies. In modern societies, due to the increasing mobility, people have more and more opportunities to interact with outgroup members. As a consequence, there have been incidents of escalating intergroup conflicts. For example, a growing number of terrorist attacks occur around the world (Roser et al., 2018), of ongoing armed conflicts in the Middle East, and of interracial killings in America (Cella and Neuhauser, 2016). In this research, we aim to investigate factors that may promote outgroup Tolerance (n.d.), that is, to be friendly and show positive attitudes to outgroup members (e.g., “sympathy or indulgence”).

Traditionally, scholars focused on individual-level theories of tolerance (Weldon, 2006), of which the most influential one is the contact hypothesis (Allport, 1954). According to the contact hypothesis, tolerance can be learned through increasing contacts with members from other groups (i.e., outgroup, Marquart-Pyatt and Paxton, 2007; Pettigrew and Tropp, 2008). Interactions with outgroup members should help people understand the perspectives of others, which lead to respect different opinions and increase tolerance (Allport, 1954; Pettigrew, 1998). Thus, according to the contact hypothesis, individuals who have contacts with outgroup members are not expected to have escalating conflicts; instead, such interactions are expected to result in reducing conflicts. In addition, this beneficial effect may not be limited to the specific group members people interact with, but may extend to members of other outgroups (indirect evidence from reducing prejudice extending to a third group, Pettigrew, 2009). Thus, intergroup interactions are expected to be conductive to outgroup tolerance.

Instead of focusing on the individual-level theories of tolerance, we sought insight from the democracy system. Democratic societies offer many opportunities for people to interact with members of different groups, such as in deliberative democracy—people of different opinions may discuss their ideas and concerned issues before coming to conclusions (Fearon, 1998; Pattie and Johnston, 2008). Through discussions, people may reveal and share information, justify their claims, which help legitimize final decisions (Fearon, 1998). Studies have demonstrated that deliberation may generate changes in opinions (Hansen, 2004; Sturgis et al., 2005) and facilitate mutual understanding and broad tolerance (Fung, 2003; Hansen, 2004; Hansen and Andersen, 2004; Hansen, 2007).

In addition, democratic societies offer opportunities for people to learn about different opinions without actual interactions. For example, people can learn different perspectives by hearing the speeches of the representatives of other groups in the media, in public, or from demonstrations. People may become more tolerant, when they are exposed to new ideas, opinions, and beliefs of other groups than when they are not (Putnam, 1993). Marcus et al. (1995) argued that the exposure to diverse opinions in mass media or publications may provide an incentive for individuals to lessen their reliance on established beliefs and increase their abilities to deal with dissenting ideas. Learning oppositional viewpoints may help people see that there is more than one side to an issue (Mutz, 2006), which is positively correlated with people’s tolerance toward disliked groups (Huckfeldt et al., 2004). Thus, in democratic societies, there may be various ways to cultivate diverse opinions and values and as a consequence people may increase tolerance and understanding of different outgroups.

However, the incidents of intergroup conflicts in democratic societies suggests that democracy is no panacea in intergroup conflicts. For example, there were 6,063 incidents of hate crimes in America in 2016; 58.9% were motivated by race bias, 21.1% by religion bias, and 16.7% by sexual orientation bias (Federal Bureau of Investigation, 2016). The violent incidents of backlash against immigrants were observed in Germany (Eddy, 2015). The fact that people in these societies use non-normative collective actions (e.g., riots, attacks) rather than normative collective actions (e.g., voting and political mobilization) raises the question of whether and how democracy may lead to people’s tolerance of others.

One possible factor contributing to the association of democracy and outgroup tolerance is democratic maturity (Inglehart, 1997; Peffley and Rohrschneider, 2003; Hegre, 2014). Mature democracy may instill people with important values (e.g., equality and justice), encourage people to understand and respect others’ views, and alleviate perceived intergroup threat (Sullivan and Transue, 1999; Kinsella and Rousseau, 2009). Perhaps as a consequence, conflicts in mature democracies are usually resolved through voicing and vote rather than riots and attacks (Crespy, 2014). In less mature democratic societies, however, people have limited freedom of expression or rights participating in politics and that may undermine social and interpersonal trust (Boyadjieva and Ilieva-Trichkova, 2015). In such societies, people are unable to achieve social equality by normative collective actions (political participation), which may cause intergroup regression and conflict (Hegre, 2014). Because there is not yet hard evidence to substantiate the causality between the democratic system characteristics and citizens’ tolerance or potential mediational processes, the first goal of this research is to fill this void by investigating (1) the causal relationship between democracy and outgroup tolerance and (2) potential mediation processes accounting for the effects of democracy on outgroup tolerance.

### Democracy: Characteristics of the System

To study the effects of democracy on outgroup tolerance, we drew insights from deliberative democracy (Habermas, 1984; Hansen, 2004), social choice theory (Riker, 1982), and intergroup emotions (Mackie and Smith, 2017). According to deliberative democracy and social choice theory, we target two characteristics of democracy, opinion sharing and voting, which have been identified essential in ensuring the integrity of democracy (Diamond et al., 1990). Voice and voting are fundamental political rights of citizens. Both of them entitle people to shape their lives according to their own choices (Welzel, 2006). We should note that in real life, the two democratic characteristics often go hand-in-hand, especially in mature democracy. That is, societies that welcome members to voice different opinions also allow members to participate in sorts of activities that may decide the future of the societies (e.g., voting). However, for conceptual clarity, we specify these two characteristics as if they were independent.

### Opinion Sharing

According to the analysis of deliberative democracy, opinion sharing has been a key feature of democracy (Cohen, 1989). Deliberative democracy focuses on democratic means, defined as people having equal access to debate, equal opportunity to introduce proposals and voice objections, and new alternatives into the discourse (Benhabib, 1994). In democratic societies, there are often a number of different channels through which citizens can express their viewpoints (Dryzek, 1990). Opinion sharing allows individuals to state their arguments, which may increase people’s knowledge and mutual understanding (Hansen, 2007). Perhaps through the increase of knowledge and mutual understanding, people may sometimes change their initial position in politic issues (Hansen and Andersen, 2004). By accessing and expressing different ideas, people might learn to tolerate and respect different ideas (Warren, 1992). Stable and robust democracy requires citizens to tolerate others’ voices and to participate in politics (Sullivan and Transue, 1999).

There are, however, contradictory incidents that may challenge the beneficial effects of opinions sharing on outgroup tolerance. In superficial intergroup interactions, negative emotions may occur due to intergroup categorization (Mackie and Smith, 2017) and when individuals have contact with outgroup members (e.g., intergroup anxiety, Stephan et al., 1999; generalized feeling of awkwardness, anxiety and apprehension, Stephan and Stephan, 1985; Stephan et al., 1999). When facing outgroup members, negative emotions are common reactions that may lead to prejudice and discrimination (Smith, 1993; Mackie et al., 2000). Outgroup members are seen as threatening and that leads to negative intergroup emotions and motivates intergroup bias (Mackie et al., 2008).

However, if opinion sharing occurs in in-depth interactions, positive emotions may occur (Spencer-Rodgers and McGovern, 2002) and help improve intergroup relations. Domestic citizens may feel curious, interested and inspired by foreigners (Spencer-Rodgers, 2001). Some certain positive emotion, such as hope, was found to contribute to decreasing intergroup conflict (Cohen-Chen et al., 2017) and negatively associated with delegitimizing perceptions of the outgroup (Halperin et al., 2008). In addition, gratitude and elevation may promote affiliative behavior (Algoe and Haidt, 2009; Layous et al., 2017), compassion may center on the wellbeing of others (Haidt, 2003), and admiration may motivate self-improvement (Algoe and Haidt, 2009).

Thus, we argue that in-depth opinion sharing in democratic societies may produce different positive emotions which have different consequences. Researchers suggested that in-depth opinion sharing may strengthen the perceptions of procedural legitimacy (Habermas, 1996), such as considering the procedure fair and trusting that the authority is concerned with their welfare (Lind et al., 1990; McFarlin and Sweeney, 1996; Watson and Angell, 2007), and consequently respond positively to the ultimate outcomes (Tyler and Lind, 1992; Folger and Cropanzano, 1998). Individuals may feel that they are entitled to voice their opinion, and when the sense of entitlement is confirmed, they may feel respected and report more positive emotions. Indeed, when group members are asked to share opinions in order to reach a decision, they express stronger positive emotions than those who are not asked (Cremer and Stouten, 2005). Therefore, we expect that participants who have opportunities to share opinions with others may report stronger positive emotions than those who do not.

We further identify one specific kind of positive emotions (i.e., other-oriented positive emotions) and explore its effects on outgroup tolerance. Other-oriented positive emotions (e.g., caring) have been found to be associated with cooperation, reciprocity, bonding (Shiota, 2014), and being benevolent toward others that significantly reduce implicit racial bias (Stell and Farsides, 2016). Other-oriented positive emotions may also trigger positive thoughts and feelings about others, which lead to doing good to others (Haidt, 2003). The studies suggested that other-oriented positive emotions generally promote interacting with others in prosocial ways (Algoe and Haidt, 2009; Layous et al., 2017). However, other positive emotions, such as happiness, have been found associated with the use of heuristics (Shiota, 2014), which may lead to increasing negative thoughts, feelings and stereotypes toward outgroups (Bodenhausen et al., 1994; Ruder and Bless, 2003; Huntsinger et al., 2009). Based on the aforementioned indirect evidence, we propose that other-oriented positive emotions (e.g., caring) may increase outgroup tolerance. In short, we hypothesize that when people are allowed to share opinions, they may have more other-oriented positive emotions, and in turn, increased tolerance toward an outgroup than when they are not allowed to share opinions.

### Voting

In addition to opinion sharing, popular participation in polity through voting is considered a crucial aspect of democracy in social choice theory (Riker, 1982). Due to the rule of “everyone counts” in democracy, voting offers individuals equal rights to make collective decisions (Lama-Rewal, 2009; Ahuja and Chibber, 2012; Carswell and De Neve, 2014) and to prevent abuse of power (Riker, 1982). Individuals who actively participate in democratic activities (e.g., petition, boycotts, and demonstration) are likely to appreciate and endorse the view that groups should have equal rights, even outgroups (Pateman, 1976; Peffley and Rohrschneider, 2014). Through voting, people learn that each person has the same right to express their own views (Banerjee, 2007). Thus, it is possible that voting may increase consciousness of equal rights. For example, members of marginalized groups report that voting is an important way to claim their rights (Carswell and De Neve, 2014); these marginalized group members typically have higher voting participation rates than privileged group members (Yadav, 1996; Palshikar and Kumar, 2004). It is possible that there is a reciprocal relation between voting and consciousness of rights. When individuals have high consciousness of rights, they vote, and through voting, their consciousness of their rights is increased.

In turn, consciousness of rights may increase outgroup tolerance. Nelson et al. (1997) found that when people are made aware of outgroups’ human rights, they express more tolerance to the disliked outgroups (e.g., allowing them to hold their own public office and demonstrations). Thus, we predict that voting will lead to increasing consciousness of rights, which in turn increases tolerance toward outgroups.

The current research examines how these two characteristics of the democratic system may affect people’s tolerance toward outgroups. Because we believe that the beneficial effects of democracy on outgroup prejudice are not limited to a specific outgroup, we targeted two outgroups. We targeted immigrants and mental patients because people have moderately negative attitudes toward these two groups (*M* = 3.07 and *M* = 3.03, ranging from 0 = *strongly dislike* to 7 = *strongly like*, Kuo, 2014, Unpublished), which may allow for contextual effects to appear. Furthermore, we targeted these two groups because they are usually stereotyped and discriminated against by others (Davies, 1998; Finch et al., 2000; Feldman and Crandall, 2007). An unfriendly social environment leads to poor mental health status in immigrants (Gee et al., 2006) and prevents mental patients from seeking treatment (Britt et al., 2008).

### Present Research

We expected that characteristics of democratic systems would be associated with outgroup tolerance. In Study 1, we used data from representative samples in 33 countries collected in 2013. Degrees of democracy were measured based on the indices of civil liberty and political participation. The index of civil liberty reflects freedom of expressing and accessing different viewpoints, which is consistent with our conceptualization of opinion sharing. Political participation indicates that people participate in democratic activities through actions such as voting. We tested whether the degree of democracy—civil liberties and political participation—was linked positively to outgroup tolerance (Hypothesis 1). In Study 2, we experimentally manipulated the two characteristics in a democratic setting: opinion sharing and voting. We expect that opinion sharing and voting may increase tolerance toward outgroups (Hypothesis 1). We also tested two mediation effects. We investigated whether opinion sharing increases tolerance toward outgroups through increasing other-oriented positive emotions (Hypothesis 2). We also investigated whether voting increases tolerance toward outgroups through increasing rights consciousness (Hypothesis 3).

## Study 1

### Method

#### Sample

We used data from the Economist Intelligence Unit’s Democracy Index (Economist Intelligence Unit, 2014) to evaluate the levels of democracy in the 33 countries included in the International Social Survey Programme (ISSP) 2013 module on National Identity. The ISSP is a multinational collaboration program that conducts social sciences surveys with diverse topics each year. The dataset includes representative samples in 33 countries from five continents, which provides a broad geographical range. The data were collected using face-to-face interviews. After removing participants younger than 18 years old (0.7%), the remaining sample included 45,070 (53.6% female) respondents aged between 18 and 112, with an average age of 47.41 (*SD* = 17.36). Missing data were handled throughout the analyses using listwise deletion. We have 44,192 responses in positive-phrased items; 44,245 responses in negative-phrased items.

#### Measures

##### Levels of democracy

The Economist Intelligence Unit collects 60 indicators grouped in five domains that measure election process and pluralism, functioning of government, political participation, civil liberties, and political culture. Because we aim to explore the effects of voting and opinion sharing on outgroup tolerance, we targeted civil liberties and political participation (see the Appendix, in the Supplementary Materials for the full survery). Civil liberties include freedom of expression and accessing different viewpoints, which reflect opinion sharing. A sample item is “Is there freedom of expression and protest?” Voting is a form of political participation, which indicates to which extent citizens actually engage in politics (e.g., voter participation/turn-out for national elections). Each score ranges from 1 (lowest degree of democracy) to 10 (highest degree of democracy). The Economist Intelligence Unit’s updates data every year. We used the democracy scores of the 33 countries in 2013, the same year in which the data were collected by the ISSP. As expected, the two indices of democracy are moderately correlated [*r*(*k* = 33) = 0.43)].

##### Tolerance toward immigrants

Tolerance toward immigrants was measured by eight items (see the Appendix, in the Supplementary Materials for full items). The items in the ISSP used a five-point scale ranging from 1 (*disagree strongly*) to 5 (*agree strongly*). Eight items loaded on two factors (negative-phrased items, Eigenvalue = 3.02, loadings > 0.52; positive-phrased items, Eigenvalue = 1.24, loadings > 0.55, *r* = 0.41). One negatively phrased example item was “Immigrants increase crime rates.”(reversed code); one positively phrased example item was “Immigrants improve society by bringing new ideas and cultures.” The subscales had acceptable reliability (*α*s > 0.68). The positive-phrased items may capture the tolerance toward immigrants, whereas the negative-phrased items may demonstrate one’s prejudice against immigrants. To evaluate the effects of democratic characteristics on outgroup tolerance and outgroup prejudice, we converted the scores so higher scores indicated *more* tolerance and *less* prejudice toward immigrants.

##### Immigration rate

Because of the conflict in Syria in 2010, some European countries may have a larger influx of immigrants than other European countries. Thus, it is important for us to control for the rate of immigrants increase or decrease from 2010 to 2013, in order to rule out the possibility that countries with decreasing immigrant inflows tend to be more tolerance toward immigrants. The data was collected by the Population Division of the Department of Economic and Social Affairs of the United Nations (United Nations, 2013). The dataset contains the rate of change of the immigrant stock by countries of destination from 2010 to 2013. Positive scores mean increasing rates of immigrants during these 3 years; negative scores mean decreasing rates of immigrants during these 3 years. The mean immigration rate is 1.51, with a range of -12.5 to 8.8.

### Results and Discussion

Due to the moderate association between civil liberty and political participation (*r* = 0.43), we tested their unique contributions to outgroup tolerance but not their interaction effects (see Baron and Kenny, 1986). Due to the data embedded in each country, we run a mixed model in the SPSS. The model estimates the fixed effects of civil liberty, political participation and immigration rate (level 2 variables) and testing effects of participants’ characteristics (age and sex, level 1 variables), while allowing the intercepts to vary across nations. Intra-class correlation coefficient in this model is 0.068 on positive items and 0.084 on negative items. In other words, 6.8 and 8.4% of the total variation in intergroup tolerance occurs due to the group level, whereas 93.2 and 91.6% due to the individual level. As shown in Table 1, the results indicated significant positive effects of civil liberty (*b* = 0.06, *SE* = 0.03, *F*[1,32.98] = 2.38, *p* = 0.02) and of political participation (*b* = 0.07, *SE* = 0.03, *F*[1,33.07] = 2.24, *p* = 0.03) on positively phrased items. The effects were less stable on negatively phrased items (for civil liberty, *b* = 0.06, *SE* = 0.03, *F*[1,33.02] = 2.02, *p* = 0.05) and for political participation (*b* = 0.05, *SE* = 0.04, *F*[1,33.09] = 1.34, *p* = 0.18). Individuals in countries with higher political participation and civil liberty were more likely to show higher immigrants tolerance captured by positive-phrased items (increasing tolerance rather than decreasing prejudice). This finding provides preliminary support for Hypothesis 1, such that people in more democratic societies showed greater tolerance toward outgroups. To replicate and further examine the causal relationship between the two democratic characteristics and outgroup tolerance, we conducted Study 2, in which we experimentally manipulated the two democratic characteristics. We tested whether the two characteristics of democracy—opinion sharing and voting—may increase people’s tolerance toward outgroups (Hypotheses 1) and whether these effects were mediated by positive other-oriented emotions and rights consciousness (Hypotheses 2 and 3).

**Table 1:** Fixed effect estimate for linear mixed model for positive and negative phrased items toward immigrants: Study 1.

Variable	Estimate	*SE*	*t*
**Positive phrased items**			
Civil liberty	0.06^∗^	0.03	2.38
Political participation	0.07^∗^	0.03	2.24
Immigrants rate	-0.001	0.01	-0.131
Age	-0.003^∗∗∗^	0.0002	-13.45
Gender (reference category: female)	-0.02^∗^	0.007	-2.10
**Negative phrased items**			
Civil liberty	0.06^∗^	0.03	2.02
Political participation	0.05	0.04	1.34
Immigrants rate	0.001	0.01	0.11
Age	-0.006^∗∗∗^	0.0002	-26.60
Gender (reference category: female)	-0.05^∗∗∗^	0.008	-6.80

^∗^*p < 0.05, ^∗∗^p < 0.01, ^∗∗∗^p < 0.001*.

## Study 2

### Method

#### Participants

We recruited one hundred students (67 females) participants at a public university in Taipei, Taiwan. Seven participants were excluded: six of them failed to follow the instructions (i.e., opinions sharing), and one participant missed over a half of the items in the questionnaire. The final sample consisted of 93 participants (63 females), ranging from 17 to 23 years old, with an average age of 18. The participants were compensated 50 NT dollars.

#### Procedures

Participants were informed that they would be participating in two unrelated research projects: the first project was concerned with the school’s new course system, and the second concerned with in attitudes toward other people. We invited participants to either share opinions (Yes or No) or vote (Yes or No) on the school’s new course system (i.e., the first project) and collected the outcome variable, tolerance toward mental patients, in the second project. The design was a 2 (voting: voting vs. non-voting) × 2 (opinion sharing: sharing vs. non-sharing) between-subjects design. Participants in all conditions first read about the school’s new course system. Next, depending on their assigned condition, participants were asked to read or share opinions (opinion sharing manipulation) and vote or not vote (vote manipulation). After completing the first project, participants were told to assist with another project by filling out several scales (e.g., tolerance toward mental patients, rights consciousness, and positive emotions). After filling out the scales, participants provided demographic information. They were debriefed, thanked, and dismissed.

#### Materials

All of the materials were in Chinese. Except for the manipulation check items, all of the scales used a six-point scale ranging from 1 (*strongly disagree*) to 6 (*strongly agree*).

##### New course system report

Participants in all conditions first read that their university is planning to convert the original semester system into a semi-quarter system (three quarters a year). In the control group (no opinion sharing, no voting), participants learned that experts would further discuss the details of the plan and decide whether to execute the plan; they were simply asked to think about the plan for 1 min. In the opinion sharing only condition, participants would read four students’ opinions (two pro and two against), and then participants were asked to write down their own opinions. In the voting only condition, participants were asked to think about the new plan for 1 min and to vote for or against the plan anonymously. In the voting and opinion sharing condition, participants read and wrote the opinions first and then voted anonymously.

After the manipulation, participants responded to a series of manipulation items. To verify the effectiveness of the voting manipulation, participants were asked to indicate at which stage the current project is: voting, expert discussion, opinion sharing, and announcement. To check the effectiveness of the opinion manipulation, participants were asked to indicate whether they were offered opportunities to read about other students’ opinions (“yes” or “no”) and share their own thoughts (“yes” or “no”).

##### Tolerance toward mental patients

The 21 items of tolerance toward mental patients were revised from the Community Attitudes toward the Mentally III scales (Taylor and Dear, 1981; see the Appendix, in the Supplementary Materials for full items). The scale was translated into Chinese and back translated into English by two Chinese–English bilingual individuals. The inconsistencies were resolved through discussion. These items were designed to assess participants’ attitudes toward people with mental illness. Twelve items loaded on a main factor (Eigenvalue = 4.98; loadings > 0.45) and were analyzed further. An example item is “we have a responsibility to provide the best possible care for the mentally ill.” Higher scores indicated more tolerance toward mental patients. The reliability was good (*α* = 0.83).

##### Rights consciousness

We developed nine items from the Universal Declaration of Human Rights (United Nations, 1948; see the Appendix, in the Supplementary Materials for full items). Eight items were loaded on one factor (Eigenvalue = 3.50; loading > 0.53) and were analyzed further. An example item is “everyone/every group is allowed to demonstrate for their claims.” Higher scores indicated higher levels of rights consciousness. The reliability was good (*α* = 0.80).

##### Positive other-oriented emotions

Three other-oriented positive emotions (i.e., joy, caring, and pleasant) were adopted from Diener et al. (1995) and were translated by previous researchers (Chien et al., 2009). The scale had acceptable reliability (*α* = 0.78).

##### Levels of involvement

In order to rule out the possibility that participants showed different levels of outgroup tolerance due to their different levels of involvement in the experiment, we measured one item of involvement. The item is “from 1 *(very sufficiently)* to 6 *(very insufficiently)*, to what extent do you sufficiently consider the new course system?”.

### Results

#### Manipulation Check Items

The opinion sharing manipulation was successful. When participants were asked to share opinions, more of them reported that they read other students’ opinions than those who were not asked (92.0% vs. 27.9%, χ^2^[1,*N* = 93] = 40.46, *p* < 0.001). The voting manipulation was also successful; more participants who voted reported that the project was in the voting stage (68.1%; 32/47) than those who did not vote (8.70%; 4/46), χ^2^(1, *N* = 93) = 34.56, *p* < 0.001. However, the manipulation check shows that our manipulation was successful but not perfect. For example, some participants in the opinion sharing and voting group chose the opinion sharing only stage rather than both opinion sharing and voting stage, probably because participants regard voting as a form of opinion expression. Nevertheless, and more importantly, voting was indicated more often in the voting condition than in the control condition, which is the crucial prerequisite for testing the effect of voting on tolerance toward the outgroup.

#### Levels of Involvement

To ensure that the manipulation did not affect the degrees of participants’ involvement, we conducted an ANOVA with opinion sharing, voting, and their interaction as independent variables and levels of involvement as the dependent variable. There were no significant differences (*p*s > 0.31).

#### Tolerance Toward Mental Patients

**FIGURE 1:**
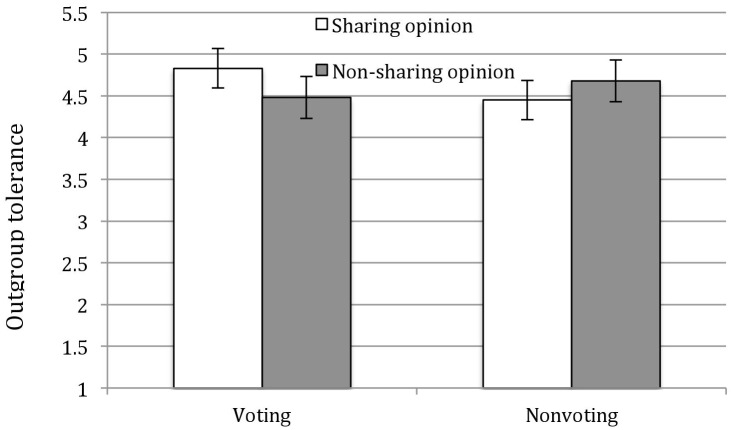
Effect of voting or without voting and opinion sharing and without sharing on outgroup tolerance toward mental patients, controlling gender and age (Study 2). Error bars represent 95% confidence intervals.

To examine whether opinion sharing and voting increased people’s tolerance to mental patients, we conducted an analysis of variance (ANOVA) with opinion sharing, voting, and their interaction as independent variables and tolerance toward mental patients as the dependent variable, controlling for participant gender and age. The main effects on tolerance toward mental patients were not significant (*p*s > 0.43). However, there was an interaction effect of opinion sharing and voting for tolerance toward mental patients, *F*(1,87) = 5.44, *p* < 0.05, η^2^ = 0.06. Specifically, among participants who voted, participants who shared opinions were found to exhibit more tolerance than those who did not (*M* = 4.83 vs. *M* = 4.48), *F*(1,87) = 4.10, *p* < 0.05, η^2^ = 0.05. In addition, among participants who shared opinions, participants who voted reported more tolerance (*M* = 4.83) than those who did not vote (*M* = 4.45, *p* < 0.05, η^2^ = 0.05 see Figure 1).

We also calculated *post hoc* power analysis with the MorePower software 6.0.4. In *post hoc* analyses, statistical power 1-β is computed as a function of significant level α = 0.05, the sample size of 93, and the effect size *F*(1,87) = 5.44, η^2^ = 0.06. According to our design (between subjects factors), the power was 0.65. Although power as high as 0.80 or higher is desirable, it is rarely seen in experimental studies and power ranging from 0.40 to 0.60 is common (Pagano, 2010). We should point out that despite our relatively lower power; we have detected a significant effect.

#### Positive Other-Oriented Emotions and Rights Consciousness

**FIGURE 2:**
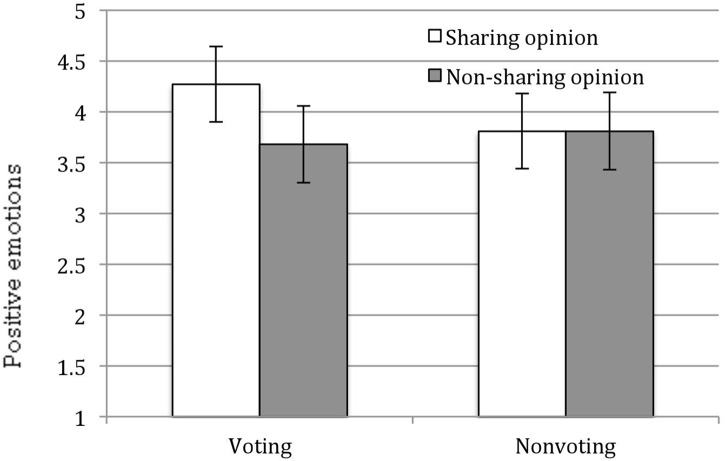
Effect of voting or without voting and opinion sharing and without sharing on positive emotions, controlling gender and age (Study 2). Error bars represent 95% confidence intervals.

To examine whether opinion sharing and voting increased people’s positive other-oriented emotions, we conducted an analysis of variance (ANOVA) with opinion sharing, voting, and their interaction as independent variables and positive other-oriented emotions as the dependent variable, controlling gender and age. We observed a trend of opinion sharing *F*(1,87) = 2.36, *p* < 0.13, η^2^ = 0.03. This effect was qualified by the interaction of opinion sharing × voting, *F*(1,87) = 2.24, *p* < 0.14, η^2^ = 0.03. Specifically, among participants who voted, participants who shared opinions were found to exhibit more positive other-oriented emotions than those who did not (*M* = 4.27 vs. *M* = 3.70), *F*(1,87) = 4.68, *p* < 0.05, η^2^ = 0.05. In addition, among participants who shared opinions, participants who voted reported marginally more positive other-oriented emotions (*M* = 4.27) than those who did not vote (*M* = 3.81, *p* = 0.09, η^2^ = 0.03 see Figure 2). However, we did not observed any significant effect for rights consciousness (*p*s < 0.37).

#### Positive Other-Oriented Emotions Mediation

Due to the interaction effect of voting and opinion sharing on outgroup tolerance, we conducted a regression analysis using a bootstrapping method of 5,000 resamples, with both opinion sharing and voting condition (coded 1) versus the other three conditions (coded -1) as a predictor, positive other-oriented emotions and rights consciousness as mediators, and outgroup tolerance as the dependent variable, gender and age as covariates. As shown in Table 2, the mediating effect of positive other-oriented emotions was significant, as indicated by the 95% confidence interval of the indirect effect [0.009, 0.179]. However, the mediating effect of rights consciousness was not confirmed. After controlling for positive other-oriented emotions, the direct effect of condition contrasted on outgroup tolerance became non-significant. Thus, the relationship between the two democratic characteristics and tolerance toward mental patients was fully mediated by positive other-oriented emotions. These results demonstrated that people who shared opinions and voted reported greater positive other-oriented emotions toward mental patients, which in turn led to greater tolerance toward the outgroup.

**Table 2:** Results of multiple mediation analyses on outgroup tolerance: Study 2.

Model	Path coefficient	*SE*	*t*
**IV to mediators (a path)**			
Opinion sharing and voting → Positive emotions	0.50^∗^	0.22	2.29
Opinion sharing and voting → Rights consciousness	0.14	0.14	0.96
**Direct effects of mediators on DV (b path)**			
Positive emotions → Outgroup tolerance	0.16^∗∗∗^	0.04	3.73
Rights consciousness → Outgroup tolerance	0.74^∗∗∗^	0.07	11.19
**Total effect of IV on DV (c path)**			
Opinion sharing and voting → outgroup tolerance	0.30^∗^	0.14	2.13
**Direct effect of IV on DV (c′ path)**			
Opinion sharing and voting → outgroup tolerance	0.13	0.09	1.37
**Model summary for DV model**			
*R^2^*	0.80		
Adjusted *R^2^*	0.63		
*F*	29.78^∗∗∗^		
**Bootstrap results for indirect effects**			
Bias-corrected confidence intervals	Lower	Upper	
Positive emotions	0.0091	1787	
Rights consciousness	-0.0867	0.2885	

*Results are presented as unstandardized coefficients. 1 = Mediation effects of voting and opinion sharing (1 = voting with opinion sharing, 0 = three other conditions) on outgroup tolerance via positive emotions, controlling gender and age. ^∗^p < 0.05, ^∗∗^p < 0.01, ^∗∗∗^p < 0.001*.

We sought to examine the causality between the democratic characteristics and outgroup tolerance and potential mediation processes. Study 2 demonstrated that democracy manipulations led to greater tolerance toward the outgroup. As expected, the positive other-oriented emotions serve as a mediator between democratic characteristics and outgroup tolerance. However, contrary to our expectations, opinion sharing and voting did not separately increase outgroup tolerance, and rights consciousness did not mediate the effect of democracy on outgroup tolerance. Taken together, the results present a framework for understanding how democratic characteristics may impact people’s tolerance toward outgroups.

## General Discussion

Despite the fact that researchers have acknowledged that democratic systems may contribute to outgroup tolerance (Sullivan and Transue, 1999; Peffley and Rohrschneider, 2003), there is a lack of solid evidence for how democratic systems may affect outgroup tolerance. In the current research, we proposed and found supporting evidence that democracy is conductive to citizens’ outgroup tolerance. Study 1 demonstrated that members in countries with higher political participation and civil liberty showed greater tolerance toward immigrants. In other words, higher levels of democracy are associated with more outgroup tolerance. Using experimental manipulations in Study 2, we found that both characteristics of democracy, namely, opinion sharing and voting, are needed to increase tolerance toward an outgroup (i.e., mental patients). The exposure to opinion sharing and voting leads to increases in positive other-oriented emotions and eventually increases outgroup tolerance. Thus, opinion sharing combined with voting appears to be a crucial contributor in increasing outgroup tolerance.

The findings from the present research contribute to our knowledge about how democratic systems may affect outgroup tolerance in several ways. First, consistent with previous research (Rohrschneider, 1996; Peffley and Rohrschneider, 2003), we found that outgroup tolerance is higher in more democratic societies (which are characterized by freedom of expressing and accessing different viewpoints, participating in democratic activities through actions). We further identified two aspects of democracy that may be beneficial to outgroup tolerance: voting and opinion sharing. Using an experimental approach, we were able to examine the causality between the two system characteristics and citizens’ tolerance. Examining specific characteristics is particularly important to understand how the democratic system may separately affect outgroup tolerance. Our findings are consistent with deliberative democracy and social choice theory: opinion sharing and voting are essential in ensuring the integrity of democracy (Diamond et al., 1990) and both of them are important for outgroup tolerance. Allowing people to express their opinions and make their decisions is a fully democratic process that reflects democratic ideology and eventually increases people’s tolerance toward outgroups. Thus, our findings suggest that the integrity of the democratic system is essential in cultivating a tolerant society.

Third, our findings suggested that the beneficial effects of democracy seem to work more robustly on increasing outgroup tolerance rather than decreasing prejudice. In Study 1, individuals in countries with higher political participation and civil liberty were more likely to show greater tolerance toward immigrants captured by positive-phrased items. Democracy encouraging people to respect societal pluralism might not necessarily change those who uphold disliking toward others, but may increase acceptance toward other groups’ viewpoints and rights. For example, the government has the responsibility to protect the rights of citizens’ political participation, even among those who may criticize the government or its leader. These experiences instill citizens with democratic norms—everyone or every group has equal rights to the claims (e.g., Gibson, 1992). As a consequence, democratic experiences may contribute to shape the society norms in which we should treat every group openly and equally regardless of our preferences.

Fourth, we observed the beneficial effects of democracy on tolerance in different outgroups. To simplify our research, we targeted one outgroup at a time, and showed that the beneficial effects of democracy were linked with tolerance toward immigrants (Study 1) and mental patients (Study 2). The findings that democratic characteristics are associated with tolerance toward immigrants (Study 1) and mental patients (Study 2) suggest that these effects are not restricted to a specific outgroup.

Fifth, our research further investigated potential mediation processes between democracy and outgroup tolerance. People experiencing caring and joy from partaking in democratic processes show more outgroup tolerance. Democracy offers unique opportunities to encourage people to seriously consider and make better decisions for their life. These experiences in democracy help individuals to feel caring, which provides a basis for creating a society with mutual love and respect. In addition, our results are consistent with recent research findings regarding the effects of positive emotions on outgroups (e.g., Stell and Farsides, 2016). Being able to claim one’s own views results in other-oriented positive emotions (e.g., Cremer and Stouten, 2005), which eventually increase outgroup tolerance at the implicit level (Stell and Farsides, 2016) as well at the explicit level (Burns et al., 2008). Our findings suggest that more work is needed to elucidate how democracy induces other-oriented positive emotions, which has implications to social harmony, social equality, and outgroup tolerance.

Finally, our results are impressive considering that the causal evidence is collected in an emerging democracy, when the beneficial effects of democracy on outgroup tolerance are expected to be weaker (see our discussion on democracy maturity). As a relatively young democracy, Taiwan has experienced a peaceful transition from authoritarian regime to democracy since the late 1980s, and also just held its first election for the provincial governor and the first presidential election in 1996. Although transition has been established in just a few decades, democracy ranking of Taiwan is relatively high in the Economist Intelligence Unit’s Democracy Index 2017 (Taiwan ranks 33 in 167 countries; Economist Intelligence Unit, 2018). Thus, citizens might effectively internalize democratic values through practicing. Our Study 2 confirmed this conjecture. Allowing people equal rights to practice democracy indeed cultivate social tolerance, which is also consistent with previous research and theories (Fung, 2003; Hansen, 2004, 2007; Hansen and Andersen, 2004; Sturgis et al., 2005; Welzel and Inglehart, 2008). Accessing different viewpoints and shaping lives by own choices may actually instill people democracy values and finally reflect on attitudes change toward outgroups (Pateman, 1976; Warren, 1992; Putnam, 1993; Marcus et al., 1995; Sullivan and Transue, 1999; Halperin et al., 2008; Peffley and Rohrschneider, 2014). Our findings suggest that encouraging and educating citizens to practice and participate in democracy may be important and effective to improve social tolerance and equality. More research is needed to explore the impact of practicing democracy in various levels of democratic countries.

Despite the encouraging evidence of democracy on outgroup tolerance in our two studies, however, rights consciousness was not found to be a mediator. Although rights consciousness predicts outgroup tolerance, voting manipulation does not significantly increase one’s rights consciousness. One possible reason for the lack of evidence between democratic practices and rights consciousness is that perhaps people in a democratic society expect to have their rights exercised, regardless of whether the procedure allows them such rights. When the procedure allows people to exercise their rights, their rights consciousness increase; when the procedure fails to provide them the opportunities to exercise their rights, they increase rights consciousness due to the lack of the opportunities. Future researchers are invited to develop creative ways to increase one’s rights consciousness.

Though not identical, the findings in our two studies complement each other. In Study 1, we found that political participation and civil liberty are moderately correlated and each predicts outgroup tolerance. In Study 2, we found that *both* opinion sharing and voting are needed to have beneficial effects on outgroup tolerance (i.e., the interaction effect). There may be several implications for these findings. First, instead of slicing democracy in pieces, we perhaps should view core elements in democracy as a whole. For example, researchers define freedom of expression and political participation as key aspects of democracy (Putnam, 1993; Inglehart, 1997; Welzel, 2006). Alexander and Welzel (2011, p. 272) wrote that “…what comes first to people’s mind when they think about democracy are the rights that give people choices in governing their personal lives, and a voice and vote to shape public life.” This finding points that mature democracy may rely on both opinion sharing and voting, and both are needed to be beneficial to outgroup tolerance.

Furthermore, it may be possible that mature democracy requires both characteristics, as evident in the moderate correlation of the two characteristics (i.e., civil liberty and political participation). According to Welzel and Inglehart (2008), mature democracy could reduce social inequality if power is vested in people: people have enough rights and freedom to express views, engage in politics, and shape public policy. Mature democracy may increase societal pluralism through encouraging and educating people to be tolerant, such as respecting others’ rights, respecting diversity of individuals and groups, and caring for every group’s well-being. Conversely, limited democracy allows the possibility of inequality. The propensity of opinion expression and political engagement are weak in limited democracy (Yeoh et al., 2013; Ho et al., 2014), and political elites usually ignores mass opinions and rights (Welzel and Inglehart, 2008). Even though citizens are allowed to voice opinions or vote, the state-imposed norms dictate citizens’ choices, and the authorities directly and indirectly control the development of social structure. We suspect that voting without opinion sharing may increase self-focused attention, which decreases outgroup tolerance. Without listening and expressing opinions, people may focus on how the final decisions affect their self-interests, which may impair intergroup relationships. For example, flawed democracies, such as an election without media freedom, undermine social and interpersonal trust (Boyadjieva and Ilieva-Trichkova, 2015), which may cause more social unrest and intergroup conflict. Furthermore, participants who are encouraged to share opinions but not allowed to participate politically may show no improvement in outgroup relationship, such as in illiberal democracies-citizens in illiberal democratic societies are constrained to participate in political activities and the authority manipulates election outcomes (Bozóki, 2017). As a result, social change and equality are hard to realize in limited democratic societies.

### Limitations

Due to the relatively small sample in Study 2 (e.g., the limited number of male participants, predominantly young adults), we should carefully generalize our findings to the population. Women and young people tend to be more emphatic than men and old people (Grühn et al., 2008; Christov-Moore et al., 2014). It may be possible that they are more likely affected by democratic characteristics than men and older people. Future research that examines the moderation effect of gender and age is indispensable to comprehensively understand the impact of democracy. Another limitation to the current research is that we only targeted two democratic characteristics. Except for opinion sharing and voting, other characteristics like demonstration and having more personal involvement (Verba et al., 1995) may also be related to intergroup attitudes. Future studies can explore other characteristics of democracy on outgroup tolerance. Methodologically, these characteristics of democracy should be specific, instead of general (e.g., levels of freedom in Freedom House and Effective Democracy Index, Alexander and Welzel, 2011), so mediation processes to outgroup tolerance may be identified. Third, correlational evidence for the beneficial effects of democracy on outgroup tolerance was gathered in the representative samples across 33 countries in Study 1 but causal evidence was gathered in only one country in Study 2. Taiwan, as a Chinese society, is embedded in Confucianism that emphasizes social hierarchy and order that ensures people obey the social rules and maintain social harmony (Lin and Ho, 2009). Although we do not have reasons to believe that our findings could not be applied to other societies, future replications examining causal evidence reflecting the impact of democratic characteristics in other cultural contexts are needed. Fourth, although we investigated tolerance with two different outgroups, immigrants and mental patients, we must be cautious about whether this effect is predictive for other outgroups; future research could continue to explore the impact of democracy on other groups. Lastly, due to the focus of our current research (democratic characteristics), we did not detail many individual-level characteristics (e.g., perceptions of outgroup threat). For example, individuals’ perceptions of outgroup threat may reduce people’s motivation to apply the democratic values (Nelson et al., 1997) and in turn decrease people’s tolerance toward outgroup (Wang and Chang, 2006). In addition, people may have different motivations to engage in political activities (Leighley and Vedlitz, 1999), such as minority group members’ motivation to obtain equal rights (Wilcox and Gomez, 1990; Stokes, 2003), dominant group members’ motivation to carry out citizen rights and duty (Carswell and De Neve, 2014). Future research incorporating system-level and individual-level characteristics is welcome. Greater understanding of the effects of democratic systems and individual characteristics is crucial in contributing to positive intergroup relationships.

## Ethics Statement

This study was carried out in accordance with the recommendations of ‘ethics guidelines for research, National Chengchi University Research Ethics Committee’ with written informed consent from all subjects. All subjects gave written informed consent in accordance with the Declaration of Helsinki.

## Author Contributions

FH investigation, writing original draft, and editing. I-CL supervision and review.

## Conflict of Interest Statement

The authors declare that the research was conducted in the absence of any commercial or financial relationships that could be construed as a potential conflict of interest.

## References

[B1] Ahuja A. Chibber P. (2012). Why the poor vote in India: “If I don’t vote, I am dead to the state”. *Stud. Comp. Int. Dev.* 47 389–410. 10.1007/s12116-012-9115-6

[B2] Alexander A. C. Welzel C. (2011). Measuring effective democracy: the human empowerment approach. *Comp. Polit.* 43 271–289. 10.5129/001041511795274931

[B3] Algoe S. B. Haidt J. (2009). Witnessing excellence in action: the ‘other-praising’ emotions of elevation, gratitude, and admiration. *J. Posit. Psychol.* 4 105–127. 10.1080/17439760802650519 19495425 PMC2689844

[B4] Allport G. W. (1954). *The Nature of Prejudice.* Cambridge, MA: Addison-Wesley.

[B5] Banerjee M. (2007). Sacred elections. *Econ. Polit. Week.* 42 1556–1562. 29358981

[B6] Baron R. M. Kenny D. A. (1986). The moderator-mediator variable distinction in social psychological research: conceptual, strategic, and statistical considerations. *J. Pers. Soc. Psychol.* 51 1173–1182. 10.1037/0022-3514.51.6.1173 3806354

[B7] Benhabib S. (1994). Deliberative rationality and models of democratic legitimacy. *Constellations* 1 26–52. 10.1111/j.1467-8675.1994.tb00003.x

[B8] Bodenhausen G. V. Kramer G. P. Susser K. (1994). Happiness and stereotypic thinking in social judgment. *J. Pers. Soc. Psychol.* 66 621–632. 10.1037/0022-3514.66.4.621

[B9] Boyadjieva P. Ilieva-Trichkova P. (2015). “Higher education and social trust: a European comparative perspective,” in *Comparative Sciences: Interdisciplinary Approaches (International Perspectives on Education and Society* Vol. 26 eds Wiseman A. W. Popov N. (Stanford, CA: Stanford University), 153–187. 10.1108/S1479-367920140000026007

[B10] Bozóki A. (2017). *Illiberal Democracy Belongs to the Hybrid Regimes.* Available at: http://www.publicseminar.org/2017/08/illiberal-democracy-belongs-to-the-hybrid-regimes/#.WfX1v2JL_1o

[B11] Britt T. W. Greene-Shortridge T. M. Brink S. Nguyen O. B. Rath J. Cox A. L. (2008). Perceived stigma and barriers to care for psychological treatment: implications for reactions to stressors in different contexts. *J. Soc. Clin. Psychol.* 27 317–335. 10.1521/jscp.2008.27.4.317

[B12] Burns K. C. Isbell L. M. Tyler J. M. (2008). Suppressing emotions toward stereotyped targets: the impact on willingness to engage in contact. *Soc. Cogn.* 26 276–287. 10.1521/soco.2008.26.3.276

[B13] Carswell G. De Neve G. (2014). Why Indians vote: Reflections on rights, citizenship, and democracy from a Tamil Nadu Village. *Antipode* 46 1032–1053. 10.1111/anti.12081

[B14] Cella M. Neuhauser A. (2016). *Race and Homicide in America, by the Numbers. U.S. News*. Available at: https://bit.ly/2tCYQBP

[B15] ChienC.-L.LiM.-C.HuangL.-L. (2009). 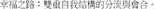 Multiple ways to subjective well-being: the divergence and convergence of double self-construals in Taiwan. *Chin. Psychol. Assoc.* 51 453–470. 10.6129/CJP.2009.5104.04

[B16] Christov-Moore L. Simpson E. A. Coudé G. Grigaityte K. Lacoboni M. Ferrari P. F. (2014). Empathy: gender effects in brain and behavior. *Neurosci. Biobehav. Rev.* 46 604–627. 10.1016/j.neubiorev.2014.09.001 25236781 PMC5110041

[B17] Cohen J. (1989). “Deliberation and democratic legitimacy,” in *The Good Polity*, eds Hamlin A. Pettit P. (Oxford: Basil Blackwell), 17–34.

[B18] Cohen-Chen S. Crisp R. J. Halperin C. E. (2017). A new appraisal-based framework underlying hope in conflict resolution. *Emot. Rev.* 9 208–214. 10.1177/1754073916670023

[B19] Cremer D. D. Stouten J. (2005). When does giving voice or not matter? Procedural fairness effects as a function of Closeness of reference points. *Curr. Psychol.* 24 203–213. 10.1007/s12144-005-1022-9

[B20] Crespy A. (2014). Deliberative democracy and the legitimacy of the European Union: a reappraisal of conflict. *Polit. Stud.* 62 81–98. 10.1111/1467-9248.12058

[B21] Davies T. (1998). *ABC of Mental Health.* London: BMJ Books.

[B22] Diamond L. Linz J. J. Lipset S. M. (1990). “Introduction: comparing experiences with democracy,” in *Politics in Developing Countries: Comparing Experiences With Democracy*, eds Diamond L. Linz J. J. Lipset S. M. (Boulder, CO: Lynne Rienner), 1–37.

[B23] Diener E. Smith H. L. Fujita F. (1995). The personality structure of affect. *J. Pers. Soc. Psychol.* 69 130–141. 10.1037/0022-3514.69.1.130

[B24] Dryzek J. S. (1990). *Discursive Democracy.* New York, NY: Cambridge University Press 10.1017/9781139173810

[B25] Economist Intelligence Unit (2014). *Democracy Index 2013.* Available at: https://bit.ly/2COtIDm

[B26] Economist Intelligence Unit (2018). *Democracy Index 2017.* Available at: https://pages.eiu.com/rs/753-RIQ-438/images/Democracy_Index_2017.pdf

[B27] Eddy M. (2015). *Violent Backlash Against Migrants in Germany as Asylum-Seekers Pour in. The New York Times.* Available at: https://nyti.ms/2OXzhqf

[B28] Fearon J. D. (1998). “Deliberation as discussion,” in *Deliberative Democracy*, ed. Elster J. (Cambridge: Cambridge University Press), 44–68. 10.1017/CBO9781139175005.004

[B29] Federal Bureau of Investigation (2016). *Hate Crime Statistics 2016.* Available at: https://ucr.fbi.gov/hate-crime/2016/topic-pages/incidentsandoffenses

[B30] Feldman D. B. Crandall C. S. (2007). Dimensions of mental illness stigma: what about mental illness causes social rejection? *J. Soc. Clin. Psychol.* 26 137–154. 10.1521/jscp.2007.26.2.137 1245639

[B31] Finch B. K. Kolody B. Vega W. A. (2000). Perceived discrimination and depression among Mexican-origin adults in California. *J. Health Soc. Behav.* 41 295–313. 10.2307/2676322 11011506

[B32] Folger R. Cropanzano R. (1998). *Organizational Justice and Human Resource Management.* Thousan Oaks, CA: Sage.

[B33] Fung A. (2003). Survey article. Recipes for public spheres: eight institutional design choices and their consequences. *J. Polit. Philos.* 11 338–367. 10.1111/1467-9760.00181

[B34] Gee G. C. Ryan A. Laflamme D. J. Holt J. (2006). Self reported discrimination and mental health status among African descendants, Mexican Americans, and other Latinos in the New Hampshire REACH 2010 initiative: the added dimension of immigration. *Am. J. Public Health* 96 1821–1828. 10.2105/AJPH.2005.080085 17008579 PMC1586129

[B35] Gibson J. L. (1992). The political consequences of intolerance: cultural conformity and political freedom. *Am. Polit. Sci. Rev.* 86 338–356. 10.2307/1964224

[B36] Grühn D. Rebucal K. Diehl M. Lumley M. Labouvie-Vief G. (2008). Empathy across the adult lifespan: longituinal and experience-sampling findings. *Emotion* 8 753–765. 10.1037/a0014123 19102586 PMC2669929

[B37] Habermas J. (1984). *The Theory of Communicative Action*, Vol. 1 Boston: Beacon Press.

[B38] Habermas J. (1996). *Between Facts and Norms: Contributions to a Discourse Theory of Law and Democracy.* Cambridge: Polity Press.

[B39] Haidt J. (2003). “The moral emotions,” in *Handbook of Affective Sciences*, eds Davidson R. J. Scherer K. R. Goldsmith H. H. (Oxford: Oxford University Press), 852–870.

[B40] Halperin E. Bar-Tal D. Nets-Zehngut R. Drori E. (2008). Emotions in conflict: correlates of fear and hope in the Israeli-Jewish society. *Peace Confl.* 14 233–258. 10.1080/10781910802229157

[B41] Hansen K. M. (2004). *Deliberative Democracy and Opinion Formation.* Odense: University Press of Southern Denmark.

[B42] Hansen K. M. (2007). The sophisticated public: the effect of competing frames on public opinion. *Scand. Polit. Stud.* 30 377–396. 10.1111/j.1467-9477.2007.00185.x

[B43] Hansen K. M. Andersen V. N. (2004). Deliberative democracy and the deliberative poll on the Euro. *Scand. Polit. Stud.* 27 261–286. 10.1111/j.1467-9477.2004.00106.x

[B44] Hegre H. (2014). Democracy and armed conflict. *J. Peace Res.* 51 159–172. 10.1177/0022343313512852

[B45] Ho L.-C. Alviar-Martin T. Leviste E. P. (2014). “There is space, and there are limits”: the challenge of teaching controversial topics in an illiberal democracy. *Teach. Coll. Rec.* 116 1–28.

[B46] Huckfeldt R. Mendez J. M. Osborn T. (2004). Disagreement, ambivalence and engagement: the political consequences of heterogeneous networks. *Polit. Psychol.* 25 65–95. 10.1111/j.1467-9221.2004.00357.x

[B47] Huntsinger J. R. Sinclair S. Clore G. L. (2009). Affective regulation of implicitly measured stereotypes and attitudes: automatic and controlled processes. *J. Exp. Soc. Psychol.* 45 560–566. 10.1016/j.jesp.2009.01.007

[B48] Inglehart R. (1997). *Modernization and Postmodernization.* Princeton: Princeton University Press.

[B49] Kinsella D. Rousseau D. L. (2009). “Democracy and conflict resolution,” in *The SAGE Handbook of Conflict Resolution*, eds Bercovitch J. Kremenyuk V. Zartman W. (London: SAGE Publications Ltd.), 475–491. 10.4135/9780857024701.n25

[B50] Lama-Rewal S. T. (2009). *Studying Elections in India: Scientific and Political Debates. South Asia Multidisciplinary Academic Journal, 3.* Available at: http://samaj.revues.org/2784 10.4000/samaj.2784

[B51] Layous K. Nelson S. K. Kurtz J. L. Lyubomirsky S. (2017). What triggers prosocial effort? A positive feedback loop between positive activities, kindness, and well-being. *J. Posit. Psychol.* 12 385–398. 10.1080/17439760.2016.1198924

[B52] Leighley J. E. Vedlitz A. (1999). Race, ethnicity, and political participation: competing models and contrasting explanations. *J. Polit.* 61 1092–1114. 10.2307/2647555

[B53] Lin L.-H. Ho Y.-L. (2009). Confucian dynamism, culture and ethical changes in Chinese societies - a comparative study of China, Taiwan, and Hong Kong. *Int. J. Hum. Resour. Manag.* 20 2401–2417. 10.1080/09585190903239757

[B54] Lind E. A. Kanfer R. Earley P. C. (1990). Voice, control, and procedural justice: instrumental and noninstrumental concerns in fairness judgment. *J. Pers. Soc. Psychol.* 59 952–959. 10.1037//0022-3514.59.5.952

[B55] Mackie D. M. Devos T. Smith E. R. (2000). Intergroup emotions: explaining offensive action tendencies in an intergroup context. *J. Pers. Soc. Psychol.* 79 602–616. 10.1037//0022-3514.79.4.602 11045741

[B56] Mackie D. M. Smith E. R. (2017). Group-based emotion in group processes and intergroup relations. *Group Process. Intergroup Relat.* 20 658–668. 10.1177/1368430217702725

[B57] Mackie D. M. Smith E. R. Ray D. G. (2008). Intergroup emotions and intergroup relations. *Soc. Personal. Psychol. Compass* 2 1866–1880. 10.1111/j.1751-9004.2008.00130.x

[B58] Marcus G. E. Sullivan J. L. Theiss-Morse E. Wood S. L. (1995). *With Malice Toward Some: How People Make Civil Liberties Judgments.* Cambridge: Cambridge University Press 10.1017/CBO9781139174046

[B59] Marquart-Pyatt S. Paxton P. (2007). In principle and in practice: learning political tolerance in eastern and western Europe. *Polit. Behav.* 29 89–113. 10.1007/s11109-006-9017-2

[B60] McFarlin D. B. Sweeney P. D. (1996). Does having a say matter only if you get your way? Instrumental and value expressive effects of employee voice. *Basic Appl. Soc. Psychol.* 18 289–303. 10.1207/s15324834basp1803-3

[B61] Mutz D. (2006). *Hearing the Other Side: Deliberative Versus Participatory Democracy.* New York, NY: Cambridge University Press 10.1017/CBO9780511617201

[B62] Nelson T. E. Clawson R. A. Oxley Z. M. (1997). Media framing of a civil liberties conflict and its effect on tolerance. *Am. Polit. Sci. Rev.* 91 567–583. 10.2307/2952075

[B63] Pagano R. R. (2010). *Understanding Statistics in the Behavioral Sciences*, 9th Edn. Australia: Wadsworth Cengage Learning.

[B64] Palshikar S. Kumar S. (2004). Participatory norm: how broad-based is it? *Econ. Polit. Week.* 39 5412–5417.

[B65] Pateman C. (1976). *Participation and Democratic Theory.* Cambridge, MA: Cambridge University Press.

[B66] Pattie C. J. Johnston R. J. (2008). It’s good to talk: talk, disagreement and tolerance. *Br. J. Polit. Sci.* 38 677–698. 10.1017/S0007123408000331

[B67] Peffley M. Rohrschneider R. (2003). Democratization and political tolerance in seventeen countries: a multilevel model of democratic learning. *Polit. Res. Q.* 56 243–257. 10.1177/106591290305600301

[B68] Peffley M. Rohrschneider R. (2014). “The multiple bases of democratic support: procedural representation and governmental outputs,” in *Elections and Democracy: Representation and Accountability*, ed. Thomassen J. (Oxford: Oxford University Press), 181–200.

[B69] Pettigrew T. (2009). Secondary transfer effect of contact: Do intergroup contact effects spread to noncontacted outgroups? *Soc. Psychol.* 40 55–65. 10.1027/1864-9335.40.2.55

[B70] Pettigrew T. Tropp L. R. (2008). How does intergroup contact reduce prejudice? Meta-analytic tests of three mediators. *Eur. J. Soc. Psychol.* 38 922–934. 10.1002/ejsp.504

[B71] Pettigrew T. F. (1998). Intergroup contact theory. *Annu. Rev. Psychol.* 49 65–85. 10.1146/annurev.psych.49.1.65 15012467

[B72] Putnam R. (1993). *Making Democracy Work: Civic Traditions in Modern Italy.* Princeton: Princeton University Press.

[B73] Riker W. H. (1982). *Liberalism Against Populism: A Confrontation Between the Democracy and the Theory of Social Choice.* Waveland: Prospect Heights.

[B74] Rohrschneider R. (1996). Institutional learning versus value diffusion: the evolution of democratic values among parliamentarians in eastern and western Germany. *J. Polit.* 58 442–466. 10.2307/2960233

[B75] Roser M. Nagdy M. Ritchie H. (2018). *Terrorism. Our World in Data.* Available at: https://ourworldindata.org/terrorism

[B76] Ruder M. Bless H. (2003). Mood and reliance on the ease of retrieval heuristic. *J. Pers. Soc. Psychol.* 85 20–32. 10.1037/0022-3514.85.1.20 12872882

[B77] Shiota M. N. (2014). “The evolutionary perspective in positive emotion research,” in *Handbook of Positive Emotions*, eds Tugade M. M. Shiota M. N. Kirby L. D. (New York, NY: Guilford Publications), 44–59.

[B78] Smith E. R. (1993). “Social identity and social emotions: toward new conceptualizations of prejudice,” in *Affect, Cognition, and Stereotyping: Interactive Processes in Group Perception*, eds Mackie D. M. Hamilton D. L. (San Diego, CA: Academic Press), 297–315.

[B79] Spencer-Rodgers J. (2001). Consensual and individual stereotypic beliefs about international students among American host nationals. *Int. J. Int. Relat.* 25 639–657. 10.1016/S0147-1767(01)00029-3

[B80] Spencer-Rodgers J. McGovern T. (2002). Attitudes toward the culturally different: the role of intercultural communication barriers, affective responses, consensual stereotypes, and perceived threat. *Int. J. Int. Relat.* 26 609–631. 10.1016/S0147-1767(02)00038-X

[B81] Stell A. J. Farsides T. (2016). Brief loving-kindness meditation reduces racial bias, mediated by positive other-regarding emotions. *Motiv. Emot.* 40 140–147. 10.1007/s11031-015-9514-x

[B82] Stephan W. G. Stephan C. W. (1985). Intergroup anxiety. *J. Soc. Issues* 41 157–176. 10.1111/j.1540-4560.1985.tb01134.x

[B83] Stephan W. G. Stephan C. W. Gudykunst W. B. (1999). Anxiety in intergroup relationships: a comparison of anxiety/uncertainty management theory and integrated threat theory. *Int. J. Int. Relat.* 23 613–628. 10.1016/S0147-1767(99)00012-7

[B84] Stokes A. K. (2003). Latino group consciousness and political participation. *Am. Polit. Res.* 31 361–378. 10.1177/1532673X03031004002 21954896

[B85] Sturgis P. Roberts C. Allum N. (2005). A different take on the deliberative poll: information, deliberation and attitude constraint. *Public Opin. Q.* 69 30–65. 10.1093/poq/nfi005

[B86] Sullivan J. L. Transue J. E. (1999). The psychological underpinnings of democracy: a selective review of research on political tolerance, interpersonal trust and social capital. *Annu. Rev. Psychol.* 50 625–650. 10.1146/annure.psych.50.1.625 15012465

[B87] Taylor S. M. Dear M. J. (1981). Scaling community attitudes toward the mentally III. *Schizophr. Bull.* 7 225–240. 10.1093/schbul/7.2.225 7280561

[B88] Tolerance (n.d.). *In Merriam-Webster Online Dictionary.* Available at: https://www.merriam-webster.com/dictionary/tolerance (accessed October 29 2018).

[B89] Tyler T. R. Lind E. A. (1992). “A relational model of authority in groups,” in *Advances in Experimental Social Psychology*, ed. Zanna M. (San Diego, CA: Academic press), 115–292.

[B90] United Nations (1948). *Universal Declaration of Human Rights.* Available at: http://www.un.org/en/universal-declaration-human-rights/

[B91] United Nations (2013). *Trends in International Migrant Stock: The 2013 Revision-Migrants by Age and Sex.* Available at: http://www.un.org/en/development/desa/population/migration/data/estimates2/estimatestotal.shtml

[B92] Verba S. Schlozman K. Brady H. (1995). *Voice and Equality: Civic Voluntarism in American Politics.* Cambridge, MA: Harvard University Press.

[B93] Wang T. Y. Chang G. A. (2006). External threats and political tolerance in Taiwan. *Polit. Res. Q.* 59 377–388. 10.1177/106591290605900305

[B94] Warren M. (1992). Democratic theory and self-transformation. *Am. Polit. Sci. Rev.* 86 8–23. 10.2307/1964012

[B95] Watson A. C. Angell B. (2007). Applying procedural justice theory to law enforcement’s response to persons with mental illness. *Psychiatr. Serv.* 58 787–793. 10.1176/appi.ps.58.6.787 17535938

[B96] Weldon S. A. (2006). The institutional context of tolerance for ethnic minorities: a comparative, multilevel analysis of western Europe. *Am. J. Polit. Sci.* 50 331–349. 10.1111/j.1540-5907.2006.00187.x

[B97] Welzel C. (2006). Democratization as an emancipative process: the neglected role of mass motivations. *Eur. J. Polit. Res.* 45 871–896. 10.1111/j.1475-6765.2006.00637.x

[B98] Welzel C. Inglehart R. (2008). The role of ordinary people in democratization. *J. Democracy* 19 126–140. 10.1353/jod.2008.0009 28922824

[B99] Wilcox C. Gomez L. (1990). Religion, group identification, and politics among American blacks. *Sociol. Anal.* 51 271–285. 10.2307/3711178

[B100] Yadav Y. (1996). Reconfiguration in Indian politics: state assembly elections, 1993–95. *Econ. Polit. Week.* 31 95–104.

[B101] Yeoh B. S. A. Leng C. H. Dung V. T. K. (2013). Commercially arranged marriage and the negotiation of citizenship rights among Vietnamese marriage migrants in multiracial Singapore. *Asian Ethn.* 14 139–156. 10.1080/14631369.2012.759746

