# Supplementary material for: Democratic Systems Increase Outgroup Tolerance Through Opinion Sharing and Voting: An International Perspective
Source: Front Psychol. 2018 Nov 13;9:2151. doi: 10.3389/fpsyg.2018.02151 (PMC6243584; doi:10.3389/fpsyg.2018.02151)
Supplement: Supplementary file 1 [file Data_Sheet_1.docx]

Appendix

The Economist Intelligence Unit measure is based on the ratings from 60 indicators grouped in five domains, the measure includes experts’ assessments and public opinion survey—mainly the World Values Survey. The domain indexes are based on the sum of the indicator scores in the domain, converted to a 0 to 10 scale, each domain has a rating on a 0 to 10 scale. Because we only used political participation and civil liberties in the study, we listed all indicators in these two domains as below.

Political participation

1. Voter participation/turn-out for national elections,

1: if consistently above 70%

0.5: if between 50% and 70%

0: if below 50%

If voting is obligatory, score 0.

2. Do ethnic, religious and other minorities have a reasonable degree of autonomy and voice in the political process?

1: Yes

0.5: Yes, but serious flaws exist

0: No

3. % of members of parliament who are women.

1: if more than 20% of seats

0.5: if 10-20%

0: if less than 10%

4. Extent of political participation. Membership of political parties and political non-governmental organizations.

1: if over 7% of population for either.

0.5: if 4% to 7%

0: if under 4%

If participation is forced, score 0

5.1 citizens’ engagement with politics.

1: High

0.5: Moderate

0: Low

5.2 If available, from World Values Survey

% of people who are very or somewhat interested in politics.

1: if over 60%

0.5: if 40% to 60%

0: if less than 40%

6.1 The preparedness of population to take part in lawful demonstrations.

1: High

0.5: Moderate

0: Low

6.2 If available, from World Values Survey

% of people who have taken part in or would consider attending lawful demonstrations.

1: if over 40%

0.5: if 30% to 40%

0: if less than 30%

7. Adult literacy

1: if over 90%

0.5: if 70% to 90%

0: if less than 70%

8.1 Extent to which adult population shows an interest in and follows politics in the news.

1: High

0.5: Moderate

0: Low

8.2 If available, from World Values Survey

% of population that follows politics in the news media (print, TW or radio) every day.

1: if over 50%

0.5: if 30% to 50%

0: if less than 30%

9. The authorities make a serious effort to promote political participation.

1: Yes

0.5: Some attempts

0: No

Consider the role of the education system, and other promotional efforts.

Consider measures to facilitate voting by member of the diaspora.

If participation is forced, score 0.

Civil liberties

1. Is there a free electronic media?

1: Yes

0.5: Pluralistic, but state-controlled media are heavily favoured. One or two private owners dominate the media.

0: No

2. Is there a free print media?

1: Yes

0.5: Pluralistic, but state-controlled media are heavily favoured. There is high degree of concentration of private ownership of national newspapers.

0: No

3. Is there freedom of expression and protest (Bar only generally accepted restrictions such as banning advocacy of violence)?

1: Yes

0.5: Minority view points are subject to some official harassment. Libel laws restrict heavily scope for free expression

0: No

4. Is media coverage robust? Is there open and free discussion of public issues, with a reasonable diversity of opinions.

1: Yes

0.5: There is formal freedom, but high degree of conformity of opinion, including through self-censorship, or discouragement of minority or marginal views

0: No

5. Are there political restrictions on access to the Internet?

1: No

0.5: Some moderate restrictions

0: Yes

6. Are citizens free to form professional organisations and trade unions?

1: Yes

0.5: officially free, but subject to some restrictions

0: No

7. Do institutions provide citizens with the opportunity to successfully petition government to redress grievances.

1: Yes

0.5: Some opportunities

0: No

8. The use of torture by the state

1: Torture is not used

0: Torture is used

9. The degree to which the judiciary is independent of government influence.

Consider the views of international legal and judicial watchdogs. Have the courts ever issued an important judgment against the government, or a senior government official?

1: High

0.5: Moderate

0: Low

10. The degree of religious tolerance and freedom of religious expression.

Are all religious permitted to operate freely, or are some restricted? Is the right to worship permitted both publicly and privately? Do some religious groups feel intimidated by others, even if the law requires equality and protection?

1: High

0.5: Moderate

0: Low

11. The degree to which citizens are treated equally under the law.

Consider whether favoured members of groups are spared prosecution under the law.

1: High

0.5: Moderate

0: Low

12. Do citizens enjoy basic security?

1: Yes

0.5: Crime is so pervasive as endanger security for large segments.

0: No

13. Extent to which private property rights protected and private business is free from undue government influence

1: High

0.5: Moderate

0: Low

14. Extent to which citizens enjoy personal freedoms

Consider gender equality, right to travel, choice of work and study.

1: High

0.5: Moderate

0: Low

15.1 Popular perceptions on human rights protection; proportion of the population that think that basic human rights are well-protected.

1: High

0.5: Moderate

0: Low

15.2 If available, from World Values Survey:

% of people who think that human rights are respected in their country.

1: if more than 70%

0.5: if 50% to 70%

0: if less than 50%

16. There is no significant discrimination on the basis of people’s race, colour or creed.

1: Yes

0.5: Yes, but some significant exceptions.

0: No

17. Extent to which the government invokes new risks and threats as an excuse for curbing civil liberties.

1: Low

0.5: Moderate

0: High

Study 1: Tolerance toward immigrants scale

Negative items.

1. immigrants increase crime rates.

2. immigrants take jobs away from people who were born in [country name].

3. [Country name]’s culture is generally undermined by immigrants.

4. [Country name] should take stronger measures to exclude illegal immigrants.

Positive items

1. Immigrants are generally good for [country name]’s economy.

2. Immigrants improve [country name] society by bringing new ideas and cultures.

3. Legal immigrants to [country name] who are not citizens should have the same rights as [country name] citizens.

4. Legal immigrants should have equal access to public education as [country name] citizens.

Study 2: Tolerance toward mental patients scale

1. The mentally ill are a burden on society.

2. The mentally ill should be isolated from the rest of the community.

3. Increased spending on mental health services is a waste of tax dollars.

4. We have a responsibility to provide the best possible care for the mentally ill.

5. More tax money should be spent on the care and treatment of the mentally ill.

6. Anyone with a history of mental problems should be excluded from taking public office.

7. A person would be foolish to marry one who has suffered from mental illness.

8. The mentally ill should be prevented from having children by a painless operation.

9. I would not want to live next door to someone who has been mentally ill.

10. The best way to handle the mentally ill is to keep them behind locked doors.

11. The mentally ill don’t deserve our sympathy.

12. Most patients in mental hospitals don’t care how they look.

Study 2: Rights consciousness scale

1. Some individuals or groups should not have the same rights as other individuals or groups.

2. Because of the gender differences, same jobs with unequal pay are acceptable.

3. Due to the limited resource, not every group’s necessities of life should be protected.

4. There is nothing wrong with some individuals or groups who have more rights in public affairs.

5. Some individuals and groups should be forbidden to express their positions.

6. Some individuals and groups’ religious and believes have better not to publicly express.

7. Due to the limited resources, not everyone should obtain full development.

8. Due to the limited resources, not everyone should obtain free development.
